# Alterations of brain network topology and structural-functional connectivity coupling in noise-induced hearing loss based on macroscopic scale

**DOI:** 10.3389/fnins.2025.1661096

**Published:** 2026-01-12

**Authors:** Aijie Wang, Xianghua Bao, Chunye Wang, Ranran Huang, Liping Wang, Minghui Lv, Guowei Zhang

**Affiliations:** 1 Department of Radiology, Yantaishan Hospital, Yantai, China; 2 Department of Occupational, Yantaishan Hospital, Yantai, China

**Keywords:** diffusion tensor imaging, graph theory analysis, noise-induced hearing loss, resting state functional magnetic resonance imaging, structure-function coupling

## Abstract

**Objective:**

To investigate the changes in the global attributes of structural connectivity (SC) and functional connectivity (FC) graph theory, as well as the coupling topological structure of the whole brain SC-FC in patients with noise-induced hearing loss (NIHL).

**Methods:**

57 NIHL patients and 55 health controls (HCs) were included; resting-state functional magnetic resonance imaging, diffusion tensor imaging, clinical data (scales, laboratory indicators) were collected. The graph theory network analysis of SC and FC, the whole-brain SC-FC coupling were performed, and a correlation analysis was employed to analyze the results in relation to the clinical data.

**Results:**

For FC, there was no significant difference in the global indices directly between groups (*P* > 0.05, FDR corrected). For SC, the normalized clustering coefficient (γ) and small-worldness (σ) of the NIHL were higher than those of the HCs (*P* < 0.05, FDR corrected). There was no significant difference in the SC-FC coupling strength of whole brain between two groups (*P* > 0.05). The graph attribute indices and coupling values of NIHL were correlated to varying degrees with the results of anxiety scale, coagulation, inflammation and biochemical indices (*P* < 0.05).

**Conclusion:**

The brain network topology structure of NIHL patients is abnormal, but the SC-FC coupling strength does not change significantly. This may provide a basis for understanding the theoretical mechanism of brain neural function remodeling and for future more detailed and diverse studies.

## Introduction

Noise-induced hearing loss (NIHL) is a progressive sensorineural hearing impairment that typically presents symmetrically in both ears, caused by long-term exposure to noise. Noise pollution has become a serious environmental issue globally, with approximately 50 million people suffering from health damage it causes each year; about 600 million workers around the world are exposed to harmful noise, making occupational NIHL as an important public health problem, accounting for 16% of the total number of hearing loss cases in the world (Chen et al., 2020). In China, occupational-NIHL has become the second most common occupational disease after pneumoconiosis, with an estimated prevalence rate exceeding 20% (Zhou et al., 2020). Occupational-NIHL can limit communication, affect attention, and may lead to psychological issues such as anxiety and depression, while also accelerating degenerative neuronal damage and the aging process (Fetoni et al., 2022; Powell et al., 2022). Previous studies have shown that the risk of dementia in elderly people with mild hearing loss is doubled, and for those with severe hearing loss, the risk is increased five-fold (Deal et al., 2017). A decline in auditory acuity may reorganize interactions between sensory and higher-order cortical areas, prompting cognitive and cross-modal processes to enhance as compensatory mechanisms for listening difficulties. This adaptation, known as cross-modal plasticity, can deplete cognitive reserves and ultimately contribute to a decline in cognitive abilities over time (Kral and Sharma, 2023).

Recent diffusion tensor imaging (DTI) studies on NIHL have shown that the Fractional Anisotropy (FA) values of structures such as the cingulum bundle and the inferior longitudinal fasciculus have decreased (Zolkefley et al., 2024). These changes in the microscopic structure are crucial for integrating auditory input with the limbic system and higher-level cognitive processing, and may constitute an important pathological basis for cognitive load, communication difficulties, memory decline, and emotional distress in NIHL patients. Therefore, systematic research beyond the auditory pathway is needed at the large-scale whole-brain level. Resting-state functional magnetic resonance imaging (rs-fMRI) is widely used in various fields of neuroscience, particularly in assessing the development of the central nervous system, exploring the pathological mechanisms of neurological and psychiatric diseases, and studying the activity characteristics of higher brain functions (Smitha et al., 2017; Sun et al., 2021; Cosío-Guirado et al., 2024). Recent studies have shown that the realization of many higher cognitive functions of the brain depends on the collaborative work between different brain regions, rather than relying on a specific brain area alone. To some extent, the occurrence of diseases is due to abnormalities in some form of connections between related brain areas. These connections within the brain can be divided into three types: structural connectivity (SC), functional connectivity (FC), and effective connectivity (EC). SC supports FC and together they form complex brain networks that support the synchronous functional activities of the brain, reflecting the integrity of neural signals. Therefore, their coupling analysis is beneficial for elucidating the constraints, maintenance, and regulatory mechanisms of structural networks on functional networks (Pan et al., 2023). In addition, Zimmermann et al. (2018) found through large-sample studies that SC and FC exhibit unique difference patterns in cognitive subjects, and the extensive cognitive behavioral patterns of the brain reveal different connection sets in SC and FC networks. Therefore, to fully understand the connectome basis of brain activity, it is necessary to combine these two patterns.

Previous studies have indicated that patients with NIHL not only exhibit abnormal brain structure (Wang et al., 2018), but also performed a decline in the stability and coordination of functional networks, and showed noticeable alterations in connectivity activity (Shin and Nam, 2023). These changes are not only related to the strength and manner of connections between internal brain regions but may also have profound effects on patients’ cognition, emotions, and daily behaviors (Thompson et al., 2022). In addition, the noise-induced stress state can activate the body’s coagulation, inflammation and oxidative metabolism, and may be related to the structural and functional changes of the brain (Arjunan and Rajan, 2020; Frye et al., 2019), and more studies are needed to clarify its cross-action and causal mechanism. Based on this, we employed rs-fMRI and DTI data to investigate the network topological properties of patients with NIHL at the whole-brain level. Our study examined the characteristics of changes in structural network and functional network coupling, as well as their clinical significance in relation to laboratory indicators. This research offers new insights into the mechanisms underlying the occurrence and progression of brain injury in NIHL patients.

## Materials and Methods

### Subjects

From 2014 to 2022, 57 patients diagnosed with NIHL by the occupational medicine department in accordance with GBZ 49–2014 “Diagnosis of Occupational Noise-Induced Deafness” (Ministry of Health of the People’s Republic of China, 2014). During the same period, 55 male healthy volunteers were selected and matched for age and education level to form the health controls group (HCs). The PTA results of HCs were less than 25 decibels. According to the study design, only binary inclusion criteria (normal hearing: yes/no) were recorded during recruitment, and the specific values were not obtained. And the HCs had no history of any noise exposure. The Hamilton Anxiety Scale (HAMA) score was used to evaluate emotional status; the noise exposure time of the NIHL, the better ear weighted speech frequency hearing threshold value from pure tone audiometry (PTA), and laboratory indicators: coagulation function [D-Dimer, Activated Partial Thromboplastin Time (APTT), Platelet (PLT)], inflammatory indicators (ESR), and biochemical indicators (HSP90α, Hcy), were collected.

Inclusion criteria: Adult males; Han ethnicity; Right-handed; Education level from primary school to university; No history of neurological or psychiatric diseases; No systemic diseases or other factors that may affect brain structure or function. Exclusion criteria: Presence of contraindications for magnetic resonance imaging, such as pacemakers or claustrophobia; Poor quality of imaging data.

This study was approved by the Ethics Committee of the Hospital (No. 2023014, Yantaishan Lun Permit). All subjects signed informed consent. We confirm that all methods were performed in accordance with the relevant guidelines and regulations.

### MRI acquisition

Using GE Discovery MR 750 3.0 T and an 8-channel head coil for T1WI 3D-FSPGR brain volume imaging, rs-fMRI, and DTI imaging examinations, patients are positioned supine, with eyes closed, earplugs inserted to protect hearing, and the head secured. Imaging parameters are as follows: T1WI 3D-FSPGR:TR 6.9 ms, TE 3.4 ms, slice thickness 1 mm, gap 0 mm, FOV 25.6 cm × 25.6 cm, matrix 256 × 256, NEX 1, flip angle 12°, the structural sequence was obtained in 4 min and 33 s. Rs-fMRI EPI:TR 2,000 ms, TE 35 ms, slice thickness 4 mm, gap 0 mm, FOV 24 cm × 24 cm, matrix 64 × 64, NEX 1, flip angle 90°, 200 time points, the fMRI sequence was obtained in 6 min and 40 s. DTI: *b*-value: *b* = 1,000, TR 5,500 ms, TE minimum, slice thickness 3.0 mm, gap 0 mm, FOV 24 cm × 24 cm, matrix 128 × 128, NEX 1,Flip angle 90°, 0 directions of diffusion gradients, the DTI sequence was obtained in 4 min and 46 s, covering the whole brain.

### Data processing

#### Pre-processing

DTI: The PANDA toolkit^1^ based on FSL software^2^ is used for preprocessing DTI data and white matter fiber tracking, which includes the following steps: ➀ Using the b0 image as a reference, perform head motion and eddy current correction. ➁ Skull stripping to remove non-brain tissues such as scalp and skull. ➂ Tracking whole-brain fibers and averaging in multiple directions. ➃ Calculating diffusion tensor indices: computing the fractional anisotropy (FA) for each voxel. ➄ Employing the deterministic linear tracking algorithm (FACT) to obtain the whole-brain white matter fiber connection set, with fiber tracking thresholds: FA ≤ 0.2 or turning angle > 35°.

Rs-fMRI: The DPABI software^3^ is used for preprocessing, which includes the following steps: ➀ Removal of the first 10 time points of the images. ➁ Temporal slice timing correction and head motion correction, with exclusion of data where head motion exceeds 2.0 mm or head rotation angle is greater than 2.0°. ➂ Spatial normalization using the DARTEL method, and resampling of voxels to a size of 3 mm × 3 mm × 3 mm. ➃ Spatial smoothing with a full-width at half-maximum (FWHM) of 4 mm. ➄ Removal of linear drift and band-pass filtering in the range of 0.01–0.08 Hz. ➅ Regression of the global brain average signal, white matter signal, cerebrospinal fluid signal, and Friston-24 head motion parameters to minimize their effects.

#### Network construction

**FIGURE 1:**
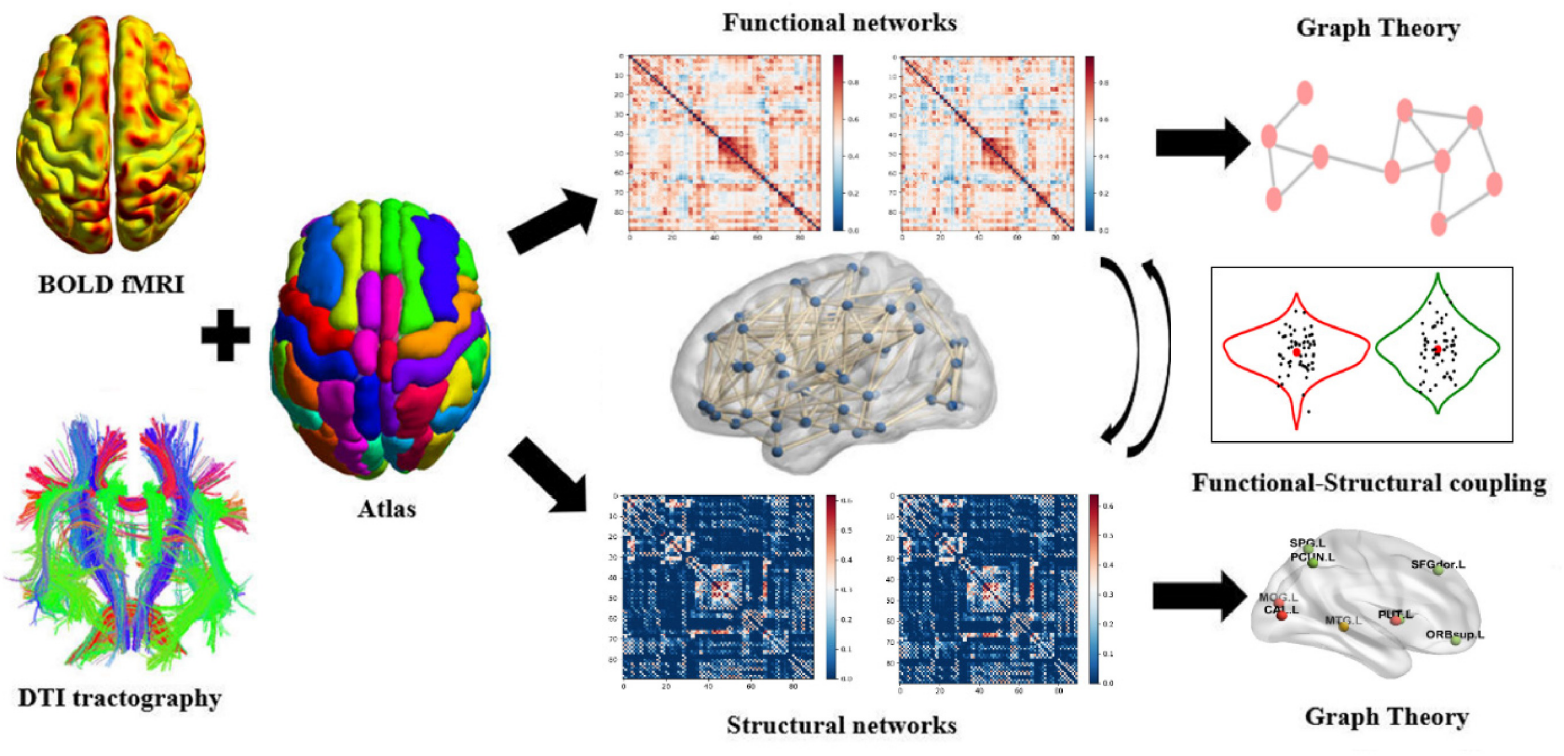
Flow chart of functional, structural network construction and whole-brain level SC-FC coupling based on rs-fMRI and DTI.

In this study, the brain was divided into 90 anatomical regions of interest (ROI) (excluding cerebellum) based on the automatic anatomical labeling algorithm (AAL90) (Tzourio-Mazoyer et al., 2002), with 45 ROI in each hemisphere as nodes. When there are at least 3 fibers between two nodes on the DTI sequence, the connection is considered to exist, and the average FA of the connecting fibers between nodes is extracted as the connecting edge of the structural network. The Pearson correlation coefficient between any two nodes is defined as the boundary of the functional network, and Fisher’s z transform is used to improve its normality. The structure and function connection matrix of 90 × 90 were constructed, respectively. Figure 1 for details (Figure 1 was generated through data processing and subsequently optimized using Microsoft PowerPoint2019).

#### Graph theory analysis

Use the GRETNA^4^ software for brain function and structure of the network graph theory analysis. The threshold values of connection sparsity of all structural and functional matrices ranged from 0.1 to 0.5, and the interval was 0.05. The global topology properties of the network include: Clustering coefficient (Cp), characteristic path length (Lp), normalized clustering coefficient (γ), normalized characteristic path length (λ), small-worldness (σ), global efficiency (Eg) and local efficiency (Eloc) (Hua et al., 2022). As the area under the curve (AUC) is an integrated scalar of a set of indicators under threshold, it provides a comprehensive evaluation indicator that is not affected by a single threshold selection and is more sensitive to changes in brain functional structure caused by disease (Qin et al., 2021). Therefore, this study further calculated the AUC of the graph at different sparsity and compared the differences between the groups.

#### SC-FC coupling analysis

The method of SC-FC coupling analysis has been described in previous studies (Chen et al., 2022). The nonzero connected edges of the SC matrix are extracted, scaled to a Gaussian distribution, and then correlated with the corresponding edges of the function matrix. SC-FC coupling values were calculated for each subject.

### Statistical analysis

The demographic, scale, and laboratory examination data were statistically analyzed using the IBM SPSS26 software. Quantitative data are expressed as the mean ± standard deviation when normally distributed, and intergroup differences were analyzed using the two independent samples *t*-test (*P* < 0.05).

A non-parametric permutation test (1,000 permutations) was used to compare between-group differences in network topology properties of two structurally and functionally related networks. By computing inter-group differences in the AUC of topological metrics across multiple sparsity thresholds, statistically significant differences are defined at *P* < 0.05 with FDR correction. Independent samples *t*-test was used to analyze whether there were between-group differences in whole-brain SC-FC coupling between the two groups. Bivariate Pearson correlation analysis was used to examine the correlation between brain structure and function indicators and clinical information. Perform curve fitting of the SC-FC coupling value with clinical indicators. And the statistical threshold was set at *P* < 0.05.

### Sensitivity analysis

In order to verify the robustness of the results, we conducted sensitivity analysis by using different brain map templates, controlling HAMA scores as confounding factors, and grouping patients with NIHL according to different degrees of weighted value of PTA whisper frequency hearing threshold.

(1)    Based on the BN246 brain atlas template,^5^ which could be divided into 246 whole brain areas, the structure-function network coupling analysis was carried out.
(2)    The HAMA score was included as a covariate to construct the structure-function network and perform the coupling analysis.
(3)    According to GBZ 49–2014 “Diagnosis of Occupational Noise Deafness” (Ministry of Health of the People’s Republic of China, 2014), patients with NIHL were divided into 40 cases of mild noise deafness group (PTA good whisper frequency threshold weighted value 26–40 dB, m-NIHL) and 17 cases of moderate and severe noise deafness group (PTA good whisper frequency threshold weighted value ≥ 41 dB, s-NIHL). The structure-function network is constructed and the coupling analysis is performed.

## Results

### Comparison of clinical data

All subjects were 35–60 years old, male, right-handed, mainly involved in drilling and welding, and the noise intensity was above 85 dB. There were no significant differences in age, years of education between the NIHL group and the HCs group, while the HAMA score of the NIHL group was higher than that of the HCs group (*P* < 0.05). The noise exposure time of the NIHL group was 5–33 years (17.32 ± 8.59 years) (Table 1).

**TABLE 1:** Comparison of general data between NIHL and HCs group (mean ± SD).

Demographic indicators	NIHL (*n* = 57)	HCs (*n* = 55)	*T*-value	*P*
Age (years)	45.54 ± 7.57	46.62 ± 7.65	*t* = 0.75	0.457
Education (years)	10.60 ± 2.12	10.91 ± 1.78	*t* = 0.85	0.399
HAMA (score)	6.12 ± 3.71	3.80 ± 1.01	*t* = -4.55	< 0.001*
Noise exposure time (years)	17.32 ± 8.59	0	–	–
PTA(dB)	37.72 ± 8.17	< 25	–	–

Two independent samples *t*-test was used for comparison between groups (*P* < 0.05). NIHL, noise induced hearing loss; HCs, health controls; HAMA, anxiety scale; PTA, pure tone threshold test. The definition of normal hearing is that the average hearing threshold of the better ear is lower than the 25 decibel hearing level specified in GBZ 49–2014.The PTA results for HCs were not recorded. The healthy control group had no history of any noise exposure.

*P < 0.05.

### Graph theory and SC-FC coupling analysis

**FIGURE 2:**
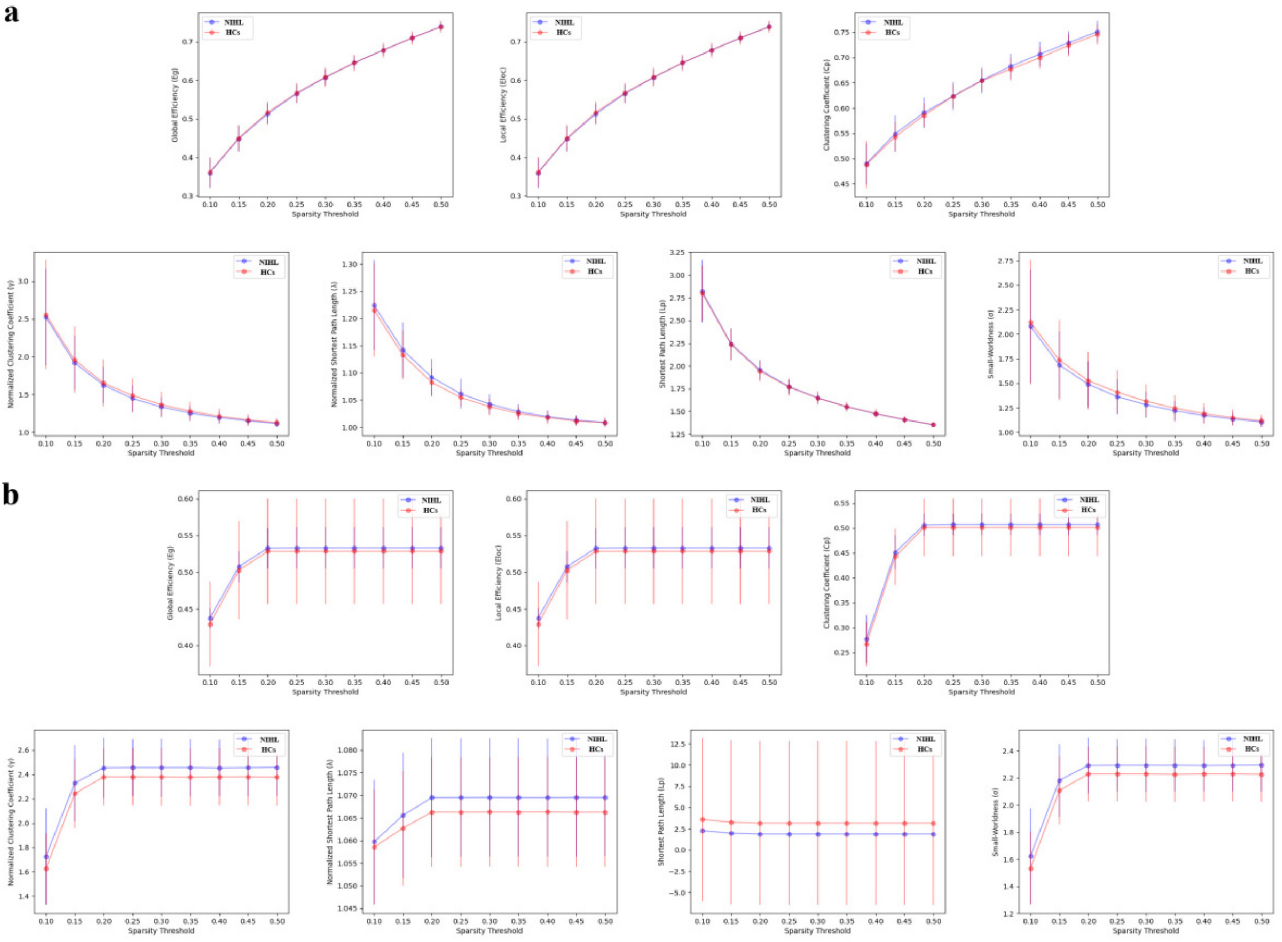
Global attribute change curves of NIHL and HCs in different sparsity threshold ranges. **(a)** FC network; **(b)** SC network. The points and error bars represent the mean and standard deviation, respectively. The blue line is the NIHL group and the red line is the HCs group. NIHL, noise induced hearing loss; HCs, health controls.

**FIGURE 3:**
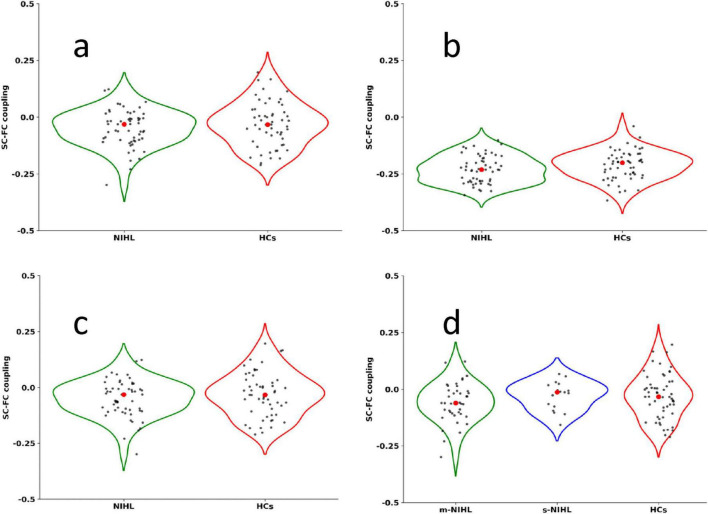
Comparison of SC-FC coupling values. **(a)** The SC-FC network coupling constructed by the AAL90 atlas. **(b)** The SC-FC network coupling constructed by the BN246 atlas. **(c)** The SC-FC network coupling constructed with HAMA score as a covariate. **(d)** The comparison of SC-FC coupling results between HCs, mild noise deafness group (m-NIHL) and moderate to severe noise deafness group (s-NIHL). NIHL, noise induced hearing loss; HCs, normal control group; SC, structural coupling; FC, functional coupling. SC-FC coupling value and *P*-values are shown in Supplementary Table 1.

Within the sparsity threshold of 0.1–0.5, both groups showed small-world topological properties of brain SC and FC (γ > 1, λ≈ 1, σ = γ / λ > 1) (Figure 2). In terms of global properties, the γ and σ values of the SC network in NIHL patients were higher than those in the healthy control group (*P* = 0.047, *P* = 0.044, after FDR correction). The Eg, Eloc, Cp, γ, λ, Lp, and σ indicators of the FC network and the Eg, Eloc, Cp, λ, and Lp parameters of the SC network did not show significant differences (*P* > 0.05, after FDR correction) (Table 2). There was no significant difference in SC-FC coupling values between NIHL group and HCs group (*P* > 0.05) (Figure 3a).

**TABLE 2:** The graph-theoretic global properties and coupling values of the whole-brain structural-functional network between NIHL and HCs.

Global properties (AUC)	NIHL	HCs	*P* (FDR corrected)
**Functional connection network**			
Eg	0.250 ± 0.009	0.250 ± 0.011	0.395
Eloc	0.250 ± 0.009	0.250 ± 0.011	0.389
Cp	0.279 ± 0.009	0.278 ± 0.008	0.139
γ	0.760 ± 0.124	0.768 ± 0.141	0.374
λ	0.495 ± 0.013	0.492 ± 0.015	0.199
Lp	0.903 ± 0.059	0.900 ± 0.059	0.348
σ	0.672 ± 0.109	0.685 ± 0.123	0.308
**Structural connection network**			
Eg	0.227 ± 0.010	0.225 ± 0.030	0.395
Eloc	0.227 ± 0.010	0.225 ± 0.030	0.438
Cp	0.204 ± 0.008	0.201 ± 0.022	0.238
γ	1.025 ± 0.118	0.989 ± 0.102	0.047*
λ	0.481 ± 0.006	0.479 ± 0.005	0.111
Lp	0.920 ± 0.049	1.503 ± 4.337	0.406
σ	0.958 ± 0.099	0.927 ± 0.088	0.044*
SC-FC coupling	-0.049 ± 0.081	-0.031 ± 0.098	0.281

NIHL, noise induced hearing loss; HCs, health controls; Eg, global efficiency; Eloc, local efficiency; Cp, clustering coefficient; γ, normalized clustering coefficient; λ, normalized characteristic path length; Lp, characteristic path length; σ, small-worldness; SC, structural connectivity; FC, functional connectivity.

*P < 0.05.

### Correlation analysis

**FIGURE 4:**
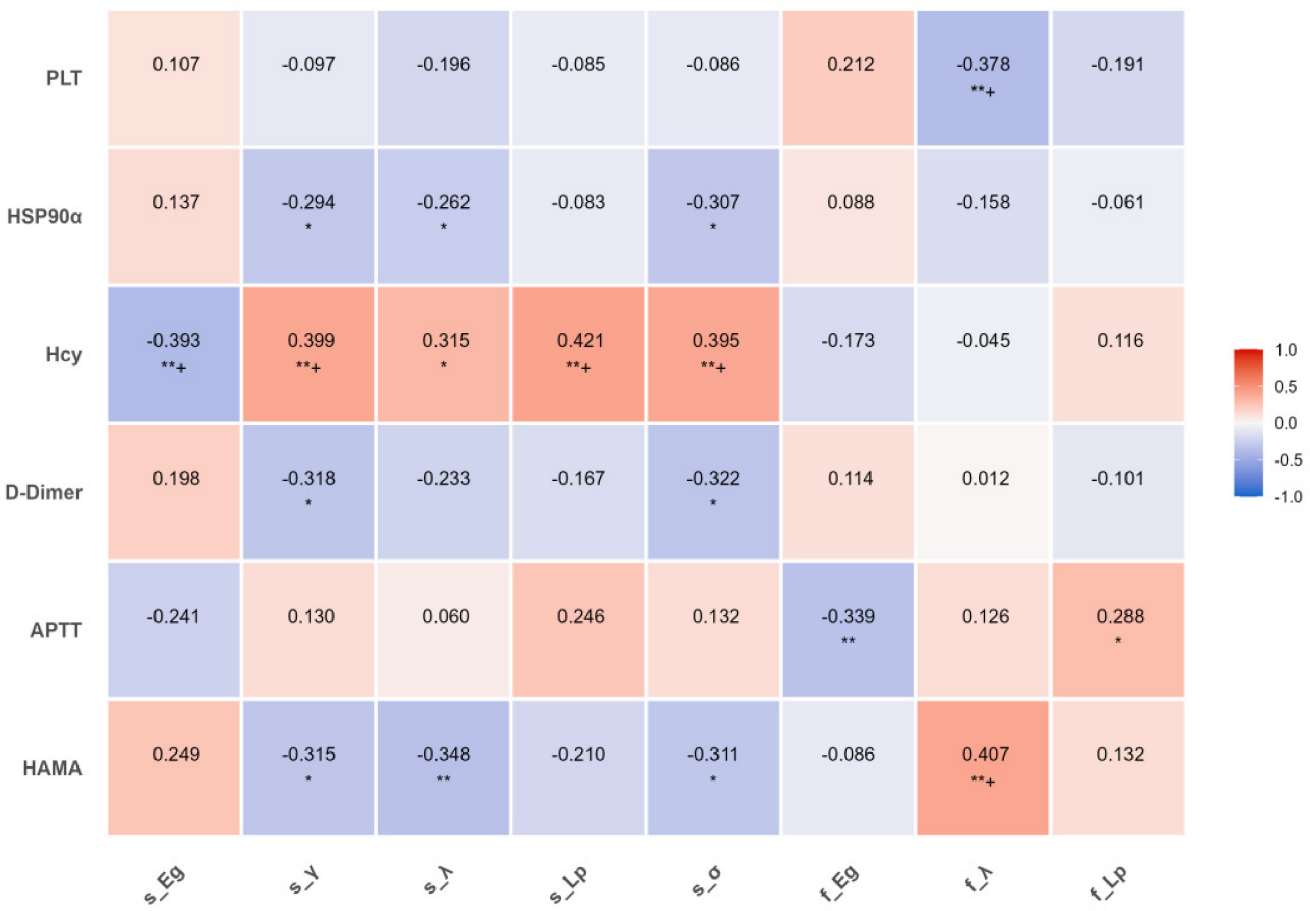
Correlation heatmap between graphical and clinical indicators. The shade of color represents the magnitude of the correlation coefficient. Red indicates a positive correlation, blue indicates a negative correlation, and the darker the color, the stronger the correlation. The numbers are Pearson correlation coefficients. *P < 0.05, **P < 0.01, and +*P* < 0.05 after FDR correction. The significance markers are located below the correlation coefficient values and are displayed only when the statistical significance is reached. *P*-values are shown in Supplementary Table 2.

**FIGURE 5:**
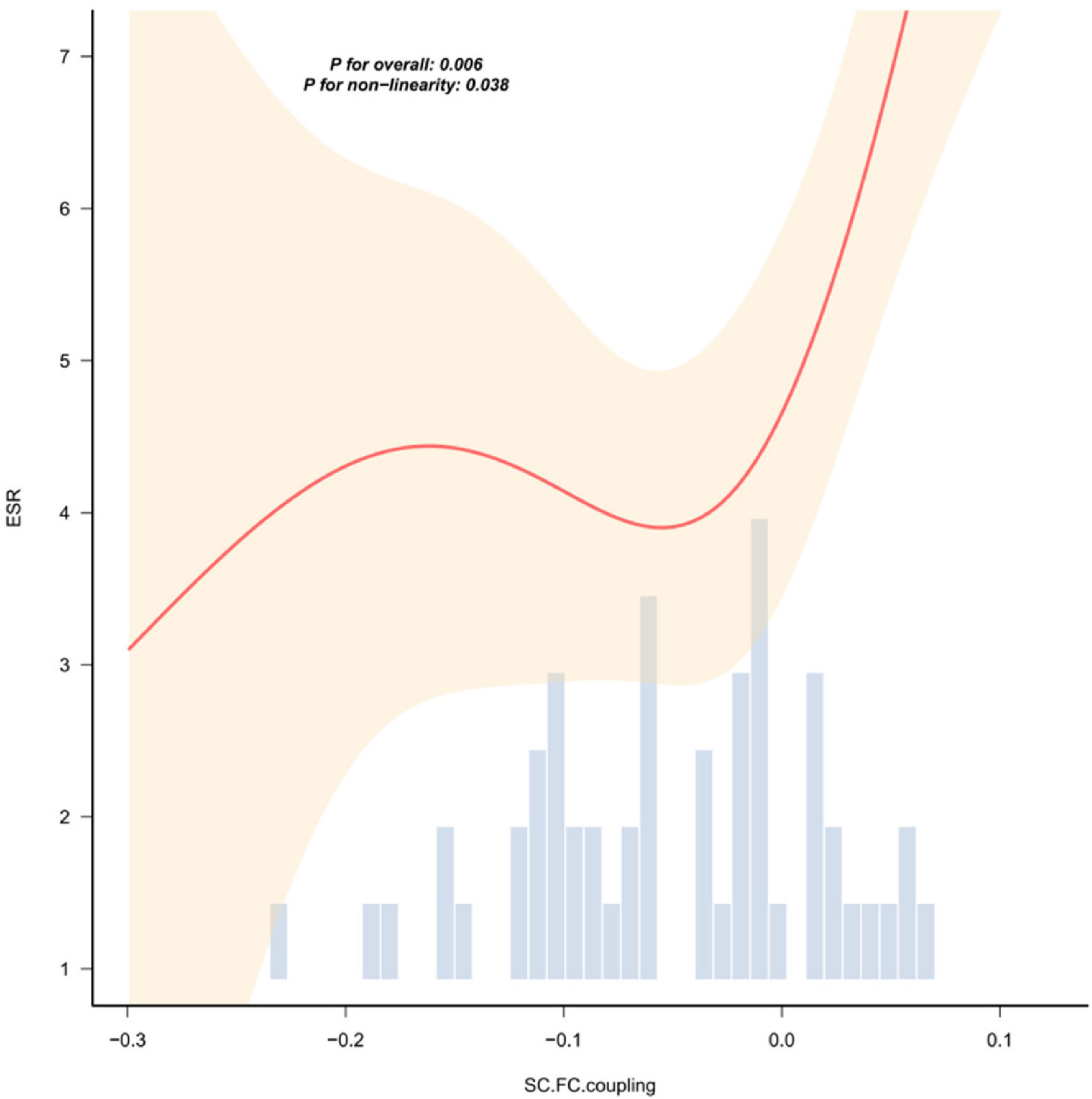
The nonlinear relationship curve between ESR and the coupling value of SC-FC. The red solid line represents the smoothed curve fitted by local weighted regression (LOESS), and the light shaded area indicates the 95% confidence interval. The bar chart below shows the distribution frequency of the SC-FC coupling values. The overall correlation is statistically significant (*P* < 0.01), and there is a nonlinear trend (*P* = 0.038). The results of the correlation analysis of other indicators without statistical significance are shown in Supplementary Figure 1.

In patients with NIHL, the global graph theory attribute indicators of the structural/functional network showed varying degrees of correlation with HAMA scores, coagulation indicators (D-dimer, APTT, PLT), and biochemical indicators (HSP90α, Hcy) (Figure 4). Curve fitting revealed a non-linear correlation between the coupling value of inflammatory indicators (ESR) and SC-FC (Figure 5).

### Sensitivity analysis result

The whole brain network constructed based on the BN246 brain map template was coupled with SC-FC and the results showed no statistical difference (Figure 3b). Due to the statistical difference in HAMA scores between the NIHL and HCs group, there was no statistical difference in the results of SC-FC coupling using them as covariates (Figure 3c). There was no significant difference in the results of SC-FC coupling between the control group, the mild noise deafness group and the moderate to severe noise deafness group (Figure 3d).

## Discussion

The functional and structural changes of the brain caused by chronic sensory deafness have been extensively studied, and deafness can lead to extensive structural and functional reorganization of the auditory center and outer brain region (Zhang et al., 2016; Tang et al., 2020). In this study, the functional activity and structural changes of the NIHL brain and SC-FC coupling were analyzed from the macro-network level, and it was found that the normalized clustering coefficients γ and small world σ of the NIHL structural network were slightly higher than those of HCs, and were correlated with anxiety and clinical laboratory indicators. Surprisingly, there was no significant difference in the SC-FC coupling analysis in NIHL patients. To our knowledge, there are few studies about using DTI and rs-fMRI to investigate the plasticity of brain activity in patients with NIHL from the perspective of structure-function coupling.

### Structural and functional network global topology attribute analysis

The small-world property of the human brain enables it to have a high clustering coefficient and short path length, which can simultaneously meet the requirements of specialization and global integration, and synchronize the integration and segregation activities of neural signals in different regions to achieve low-cost and efficient information transmission (Sporns and Zwi, 2004). Both healthy and diseased brains have small-world properties, which is an important concept in neural network science (Bassett and Bullmore, 2017). Studies have shown that the topology of brain networks changes during normal development and aging, in different diseases and even in different types of hearing impairment (Liao et al., 2017), such as senile hearing loss (Guan et al., 2022), sudden sensorineural deafness (Hua et al., 2022) and congenital hearing loss (Cui et al., 2022a).

We applied graph theory analysis and small-world network to better understand the neuroplasticity in the chronic course of NIHL. The results showed that there was no statistical difference in the global attributes of the functional network, but the normalized clustering coefficient γ and small-world attribute σ in the structural network were higher than those in HCs, suggesting that the brain structural network in patients with NIHL underwent cross-remodeling to maintain the stability of the functional network. The architecture of structural connections ensures coherence and synchronization of neuronal co-activation patterns (Suárez et al., 2020). This is consistent with previous findings that senile deafness is also a progressive bilateral high-frequency sensorineural hearing loss. Xu et al. (2024) found that in the senile deafness Lp and λ increased and Eg decreased in the low-order functional network constructed by Pearson correlation, suggesting that the global information processing ability was limited, and the change in topological properties of senile deafness may be related to the decline in cognitive level (Wang et al., 2024). A functional network study of long-term unilateral hearing loss (UHL) showed that the UHL brain network had almost the same path length (λ≈1) and a higher clustering coefficient (λ > 1) than the normal hearing group (Zhang et al., 2018), which is similar to our findings. However, the structural brain networks of NIHL patients also showed higher small-world properties, suggesting more complex neural remodeling. Zou et al. (2021) examined the white matter network of 145 cases of sudden sensorineural hearing loss (SSNHL) and found that the clustering coefficient, local efficiency, global efficiency and small-world attribute decreased, while the feature path length increased. Our results are inconsistent with the reduced global network connectivity in SSNHL patients, which is thought to be related to the specific onset and development of NIHL disease. Ponticorvo et al. (2022) performed a graph theory analysis of the functional and structural network of 52 cases of age-related hearing loss (HL) and found no significant changes in the global attributes, which they believe that it may be related to the fact that sensory deficiency in the early stage of HL has not changed the overall structure of large-scale networks. Congenital sensorineural hearing loss (SNHL) is generally a preverbal deafness. Cui et al. (2022b) found that there were no statistical differences in the small-world properties and network efficiency parameters of structural and functional networks in the SNHL group. It is concluded that cross-modal recombination after hearing loss is mainly caused by the recombination of existing connections rather than the recombination of generated connections. Considering the younger age of the participants, the stability of global topological properties may be based on maximum neural plasticity during critical developmental periods. These differences indicate that brain network remodeling patterns are not consistent across different types of hearing loss, suggesting that brain network topology indicators may be used as neuroimaging markers for different types of hearing loss.

### SC-FC coupling analysis

Exactly how the anatomy of the brain gives rise to a range of complex functions is still not fully understood, and a growing body of research has shown that the correlation between structure and the strength of functional connections is not perfect (Fotiadis et al., 2024); for example, individuals with schizencephaly or corpus callosum dysplasia may have bilateral functional connections even in the absence of large associative fibers connecting the two hemispheres (Uddin, 2013). In addition, the relationship between functional and structural connectivity changes with age, and regional SC-FC coupling may be stronger in the elderly, suggesting that healthy structural architecture prevents cognitive decline by maintaining functional communication between regions (Zimmermann et al., 2016).

Studies have found that the spatial distribution of SC-FC coupling is closely related to the functional level of the brain and is an important neural basis for advanced cognitive ability. And the direct correlation and coupling between the anatomical structure of the brain and functional activities is not fixed, but adjusted with task requirements, developmental stages and pathological states (Baum et al., 2020; Wang et al., 2015). In patients with small cerebral vascular disease (CSVD), decreasing SCN-FCN coupling within the cognitive control network was associated with deficits in general cognition, processing speed, and greater apathy (Tay et al., 2023). Abnormal SC-FC coupling is also associated with many neuropsychiatric disorders, and changes in the Structural Decoupling Index (SDI) in Alzheimer’s disease patients are significantly associated with cognitive ability, which possibly reflected changes in the hierarchical organization pattern of the brain from sensorimotor areas to higher cognitive areas (Sun et al., 2024). In addition, it has been proved that transcranial magnetic stimulation (TMS) targets to regulate the structural and functional coupling of the frontal lobe-limbic system in patients with depression, and its efficacy is positively correlated with the degree of coupling recovery (Noda et al., 2018). Therefore, the study of SC-FC coupling cannot only clarify the mechanism of the disease, provide objective indicators for the early diagnosis and classification of the disease, but also dynamically monitor and optimize the regulatory neural parameters, and provide directions for the development of protective drugs.

Although SC-FC coupling was performed at the macroscopic level, no significant differences were observed between the NIH and HCs groups. However, we acknowledge that the absence of statistically significant differences does not conclusively prove that SC-FC coupling is completely unaffected in NIHL. This null result may reflect limitations in statistical power, the relative insensitivity of overall coupling indicators, or the fact that although there is structural remodeling, functional communication has been truly preserved. If functional communication is indeed maintained, our results are consistent with effective neural plasticity compensation and functional network reorganization after noise-induced structural damage (Huang et al., 2024a,b). The potential mechanisms underlying this retention may include increased reliance on indirect multisynaptic pathways, recruitment of regional detours or alternative paths in partially damaged networks, and utilization of parallel or potential circuit-level redundancies by activating circuits that bypass damaged connections (Feng et al., 2024). These adaptive processes will enable the brain to maintain effective information transmission in the presence of major structural path impairments. Future studies will require larger sample sizes and more sensitive, regionally specific methods to distinguish these possibilities and confirm the existence and exact nature of the compensation mechanisms in NIHL.

Furthermore, the research results indicate that the coupling values of the SC-FC network in patients with NIHL are mostly negative, which is inconsistent with the previous view that functional connectivity depends on the connectivity of white matter fiber tracts (Damoiseaux and Greicius, 2009). This pattern does not mean that there is no structural-functional relationship, but may be related to various mechanisms such as research methods, neurophysiology, etc.: (i) Functional connections between cortical regions are usually mediated by indirect multi-synaptic pathways or shared inputs rather than direct single-synaptic white matter tracts, thereby generating strong FC in the absence of a corresponding strong FA-weighted SC (Honey et al., 2009; Wu et al., 2023; Liao et al., 2024). (ii) The AAL90 atlas excludes deep white matter and regions with crossed fibers, further reducing the direct SC-FC correspondence. (iii) In the entire adult life cycle and various clinical conditions (mild cognitive impairment, severe depression, and tinnitus), when using the same fiber tensor weights and threshold strategies, the negative coupling phenomenon occurs systematically (Chen et al., 2022; Zamani Esfahlani et al., 2022; Zhao et al., 2024).

### Correlation analysis

Recent DTI studies have shown that the FA values of the inferior longitudinal fasciculus (ILF) and the cingulum bundle in patients with NIHL are reduced (Zolkefley et al., 2024). The ILF constitutes the main ventral pathway connecting the auditory association cortex with the temporal lobe, parietal lobe, and limbic regions, while the cingulum bundle provides dorsal connections between the auditory cortex, prefrontal regions, and the limbic structures that are crucial for emotion regulation. These structural changes in the fiber bundles may promote compensatory reliance on local and intrahemispheric circuits, leading to increased cognitive load and anxiety distress, which is consistent with the results showing a correlation between the graph-theoretical properties and HAMA scores that we obtained.

Animal experiments have confirmed that noise exposure can induce abnormal brain functions through a cascade reaction of “local inflammation—oxidative stress—central remodeling”: On the one hand, long-term noise stimulation significantly upregulates the expression of pro-inflammatory cytokines in key regions such as the auditory cortex and limbic system of the brain, and increases the levels of oxidative stress markers, directly triggering central stress responses and exacerbating lipid peroxidation damage, ultimately disrupting the stability of cortical neural circuits and inducing neuroinflammation and abnormal synaptic plasticity (Shukla et al., 2020). In the mouse model of noise-induced deafness, the massive secretion of tumor necrosis factor (TNF) after macrophage activation not only initiates an inflammatory chain reaction in the auditory cortex but also participates in the occurrence of anxiety-like behaviors by regulating the hypothalamic-pituitary-adrenal (HPA) axis, revealing the central comorbidity mechanism of “auditory damage—emotional abnormalities” (Peng et al., 2023). On the other hand, the systemic physiological disorders caused by noise exposure can further amplify the central damage effect. Toukh et al. (2014) found that long-term noise exposure can lead to a hypercoagulable state and elevated cortisol levels in rats, and this peripheral physiological imbalance will affect the integrity of white matter structure in the brain through the blood-brain barrier and disrupt the coordination of the brain functional network.

Neurophysiological studies have further clarified that noise stimulation can directly activate the limbic system (amygdala, hippocampus, etc.) and trigger neuroendocrine disorders and abnormal emotional responses by overexciting the hypothalamic-pituitary-adrenal (HPA) axis (Toukh et al., 2014). This is consistent with the results observed in this study. The small-world characteristics and clustering coefficient of the brain network are significantly correlated with inflammation, coagulation function, and metabolic indicators. Existing evidence indicates that noise exposure can induce systemic inflammation, oxidative stress, and neuroendocrine disorders, leading to coordinated abnormalities in brain network topology and functional remodeling (Hahad et al., 2022). It should be emphasized that the causal relationship of this pathway still needs to be further verified through more translational medical research (such as multimodal imaging—molecular biology combined detection, longitudinal follow-up) to identify key regulatory targets.

### Limitation and prospect

This study still has some limitations that need to be taken into account. First, our research subjects were only male, which although reflecting the gender ratio characteristics of the selected noise-exposed workers’ occupational population, limits the applicability of our research results to the female group. And in the healthy control group, there were no specific pure tone audiometry (PTA) values available, which prevented a more precise quantitative comparison between the groups. Second, the original acquisition parameters of DTI non-isotropy may have limited sensitivity in detecting subtle white matter changes, especially in areas with complex fiber crossings, which may underestimate the degree of true auditory cortex and basal ganglia coupling changes in NIHL (Jones et al., 2013). Third, our focus on the macroscopic level of SC-FC coupling throughout the brain did not consider regional differences (such as auditory or cross-modal networks), which may mask the NIHL-specific reorganization phenomena (Chen et al., 2022).

To address the aforementioned limitations of the research, future studies should employ larger sample cohorts with greater diversity to verify the generalizability and robustness of the results of this study. At the same time, more rigorous DTI protocols (such as multi-shell acquisition, isotropic voxels) should be adopted, combined with detailed analysis at the network sub-region level, dynamic functional connectivity modeling, and multimodal techniques such as transcranial magnetic stimulation (TMS)/electroencephalogram (EEG), to more accurately reveal the neuro-pathological remodeling characteristics and temporal-spatial dynamic patterns of the brains of patients with chronic occupational NIHL.

## Conclusion

In light of the aforementioned findings, we consider: (1) The enhancement of the global attributes of the structural network may serve as the foundation for maintaining the functional network, thereby ensuring the stability of SC-FC coupling throughout the brain. (2) The alterations in brain network topology are associated with oxidative stress and metabolic activities. These findings contribute to a more comprehensive understanding of the brain network basis of neural function changes in NIHL patients, and provide crucial insights for further investigating the neuropathological mechanisms of brain plasticity.

## Data Availability

The raw data supporting the conclusions of this article will be made available by the authors, without undue reservation.

## Footnotes

1
http://www.nitrc.org/projects/panda

2
https://fsl.fmrib.ox.ac.uk/fsl/fslwiki/

3
http://www.rfmri.org/dpabi

4
http://www.nitrc.org/projects/gretna/

5
http://atlas.brainnetome.org
