# Supplementary material for: Alterations of brain network topology and structural-functional connectivity coupling in noise-induced hearing loss based on macroscopic scale
Source: Front Neurosci. 2026 Jan 12;19:1661096. doi: 10.3389/fnins.2025.1661096 (PMC12833366; doi:10.3389/fnins.2025.1661096)
Supplement: Supplementary file 1 [file Data_Sheet_1.pdf]

supplementary materials

Supplementary table 1

Supplementary table in Figure 3: Comparison of measurement of SC-FC coupling values.

	SC-FC coupling values			P values
	HCs	NIHL		
a	-0.031±0.098	-0.049±0.081		0.281
b	-0.212±0.064	-0.230±0.060		0.118
c	-0.031±0.098	-0.049±0.081		0.281
d	0.031±0.098	-0.058±0.087(m-HIHL)	-0.028±0.061(s-NIHL)	0.296

(a) the SC-FC network coupling constructed by the AAL90 atlas.(b) the SC-FC network coupling constructed by the BN246 atlas. (c) the SC-FC network coupling constructed with HAMA score as a covariate. (d) the comparison of SC-FC coupling results between HCs, mild noise deafness group (m-NIHL) and moderate to severe noise deafness group (s-NIHL). NIHL: noise induced hearing loss; HCs: normal control group; SC: structural coupling; FC: functional coupling.

Supplementary table 2

Supplementary table in Figure 4: Correlation between graph theoretic indicators and clinical indicators

Graph_Theory_Variable	Clinical_Variable	Correlation	P_Value	FDR_P_Value	Significant_after_FDR	FDR_Mark
f_λ	HAMA	0.407	0.001657	0.02366	TRUE	[FDR]
f_Lp	APTT	0.288	0.029763	0.089288	FALSE	
f_λ	PLT	-0.378	0.00371	0.029682	TRUE	[FDR]
f_Eg	APTT	-0.339	0.009957	0.059742	FALSE	
s_λ	HAMA	-0.348	0.007928	0.054365	FALSE	
s_λ	HSP90α	-0.262	0.049169	0.138829	FALSE	
s_γ	Hcy	0.399	0.002081	0.02366	TRUE	[FDR]
s_σ	Hcy	0.395	0.002354	0.02366	TRUE	[FDR]
s_Eg	Hcy	-0.393	0.002465	0.02366	TRUE	[FDR]

s_Lp	Hcy	0.421	0.001119	0.02366	TRUE	[FDR]
s_σ	D-Dimer	-0.322	0.014444	0.067845	FALSE	
s_γ	D-Dimer	-0.318	0.016056	0.067845	FALSE	
s_γ	HAMA	-0.315	0.017018	0.067845	FALSE	
s_λ	Hcy	0.315	0.017041	0.067845	FALSE	
s_σ	HAMA	-0.311	0.018375	0.067845	FALSE	
s_σ	HSP90α	-0.307	0.020205	0.069273	FALSE	
s_γ	HSP90α	-0.294	0.026238	0.083963	FALSE	

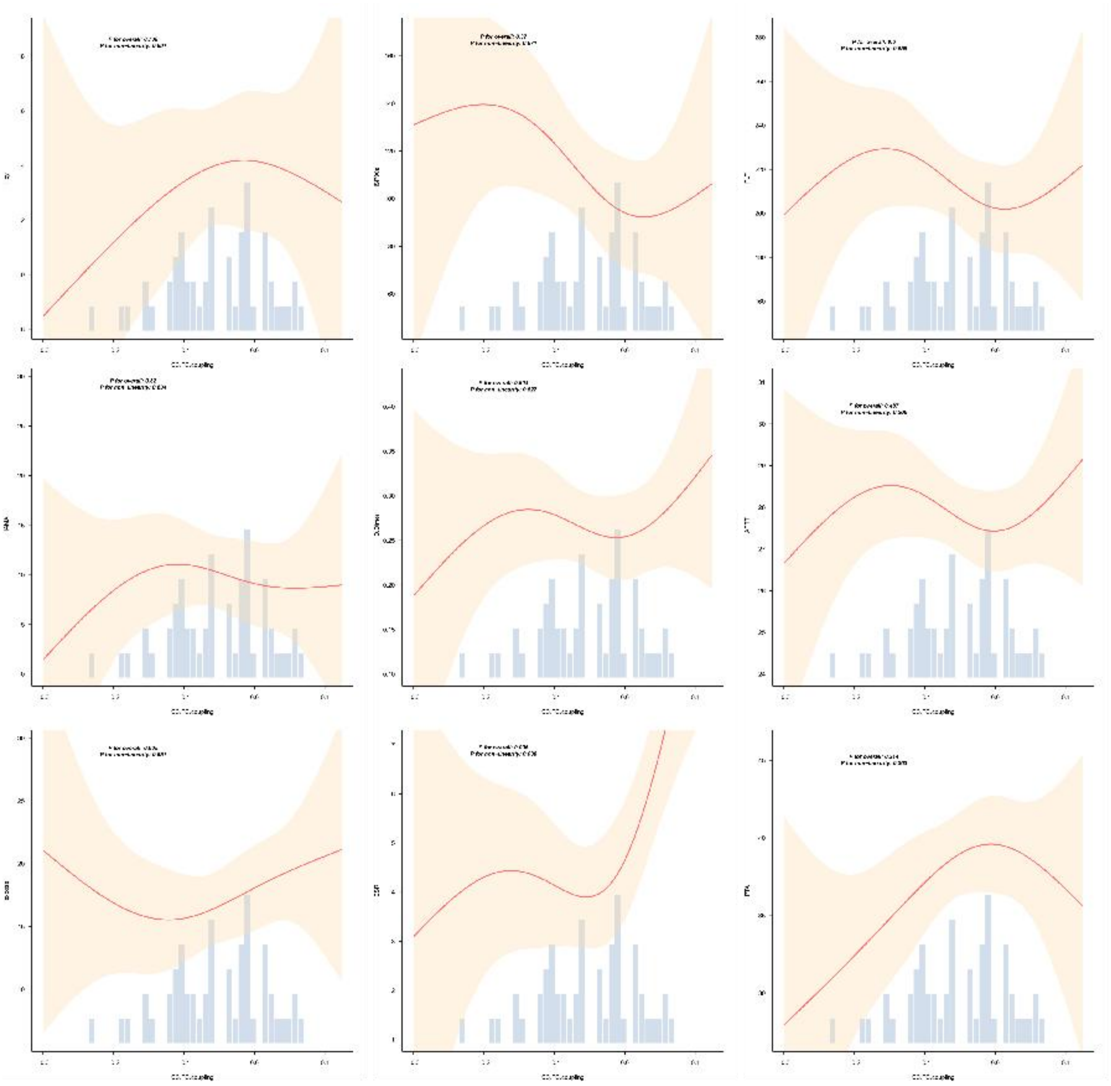

Supplementary Figure 1: The clinical indicators that were fitted with the coupling values of SC-FC

in a curve were: Hcy, HSP90 α , PLT, HAMA, D-Dimer, APTT, years of exposure, ESR, PTA.
